# Contemporary use trends and effect on survival of pelvic lymph node dissection for non-muscle-invasive bladder cancer

**DOI:** 10.3389/fsurg.2022.961430

**Published:** 2022-08-11

**Authors:** Yaxiong Tang, Kan Wu, Xiang Li

**Affiliations:** Department of Urology, Institute of Urology, West China Hospital, Sichuan University, Chengdu, China

**Keywords:** non-muscle-invasive bladder cancer (NMIBC), radical cystectomy (RC), pelvic lymph node dissection (PLND), overall survival (OS), survival analyisis

## Abstract

**Background:**

Patients diagnosed with non-muscle-invasive bladder cancer (NMIBC) who are at a very high risk of disease progression and failure of Bacillus Calmette-Guerin treatment are recommended to undergo immediate radical cystectomy (RC). The role and optimal degree of pelvic lymph node dissection (PLND) during RC for NMIBC patients, however, have not been well investigated.

**Patients and methods:**

The Surveillance, Epidemiology, and End Results (SEER) database was used to identify patients. Overall survival (OS) was assessed with the Kaplan–Meier technique. Multivariable Cox regression analysis was conducted to determine independent factors of OS.

**Results:**

A total of 1,701 patients were identified in the SEER database from 2004 to 2015. Any level of PLND (>0 lymph nodes examined) was performed in 1,092 patients (64.2%). The median number of lymph nodes examined was 8 (interquartile range, 0–20) in T1, 0 (interquartile range, 0–11) in Ta, and 0 (interquartile range, 0–14) in Tia patients. Compared with non-PLND, any level of PLND improved OS in T1 but not in Ta or Tis patients. Compared to limited (1–9 lymph nodes examined) and non-PLND, extensive PLND (lymph nodes examined ≥10) resulted in better OS only in T1 patients (all *p* < 0.001, adjusted significance level = 0.017). PLND was identified as a independent protective factor for OS.

**Conclusion:**

Based on the SEER database, we found that PLND during RC led to better OS and extensive PLND was associated with better OS in T1 but not in Ta or Tis patients. The implementation of PLND was insufficient both in population proportions and scope.

## Introduction

Bladder cancer (BC) is the tenth most common cancer worldwide, with approximately 573,000 new cases and 213,000 deaths estimated in 2020 (1). Non-muscle-invasive bladder cancer (NMIBC), which encompasses stages Ta, Tis, and T1, accounts for 75% of bladder cancer cases (2). Transurethral resection of the bladder tumor (TURBT) and Bacillus Calmette-Guerin (BCG) intravesical infusion are presently the main treatments for NMIBC, with radical cystectomy (RC) advised for patients at a very high risk of progression according to the 2021 European Association of Urology (EAU) grading model and who have failed BCG therapy (3). Even though RC with pelvic lymph node dissection (PLND) is now the standard therapy for patients with muscle-invasive bladder cancer (MIBC) (4, 5), the role and extent of PLND during RC for NMIBC patients are still controversial (6–11).

Lymph node metastasis is considered to be the most common metastasis in BC (12). Although patients with NMIBC who underwent RC had a decreased rate of lymph node metastasis, this is stage-specific and can be as high as 40% in T1 patients (9). T1 tumors also have poor staging accuracy *via* TURBT, with 27%–51% of patients being upstaged to MIBC at RC after RC (13, 14), for whom RC with PLND should be the standard treatment. With these findings, PLND may be required for some NMIBC patients. On the one hand, PLND may have a direct therapeutic impact by eradicating micrometastasis, perhaps reducing the risk of recurrence. On the other hand, PLND can enable patients to be staged more accurately, allowing for better identification of patients who may benefit from adjuvant therapy. However, because of the increased surgical difficulty, longer operative time, and higher perioperative morbidity rate associated with PLND (15, 16), it is vitally important to carefully consider whether and to what extent PLND should be performed during RC for NMIBC patients.

In this study, we used the National Cancer Institute's Surveillance, Epidemiology, and End Results (SEER) database to obtain data on patients who were diagnosed with NMIBC and underwent RC to explore the contemporary use trends and the effect on survival of PLND in patients diagnosed with NMIBC who underwent RC.

## Patients and methods

### Patients

**Figure 1:**
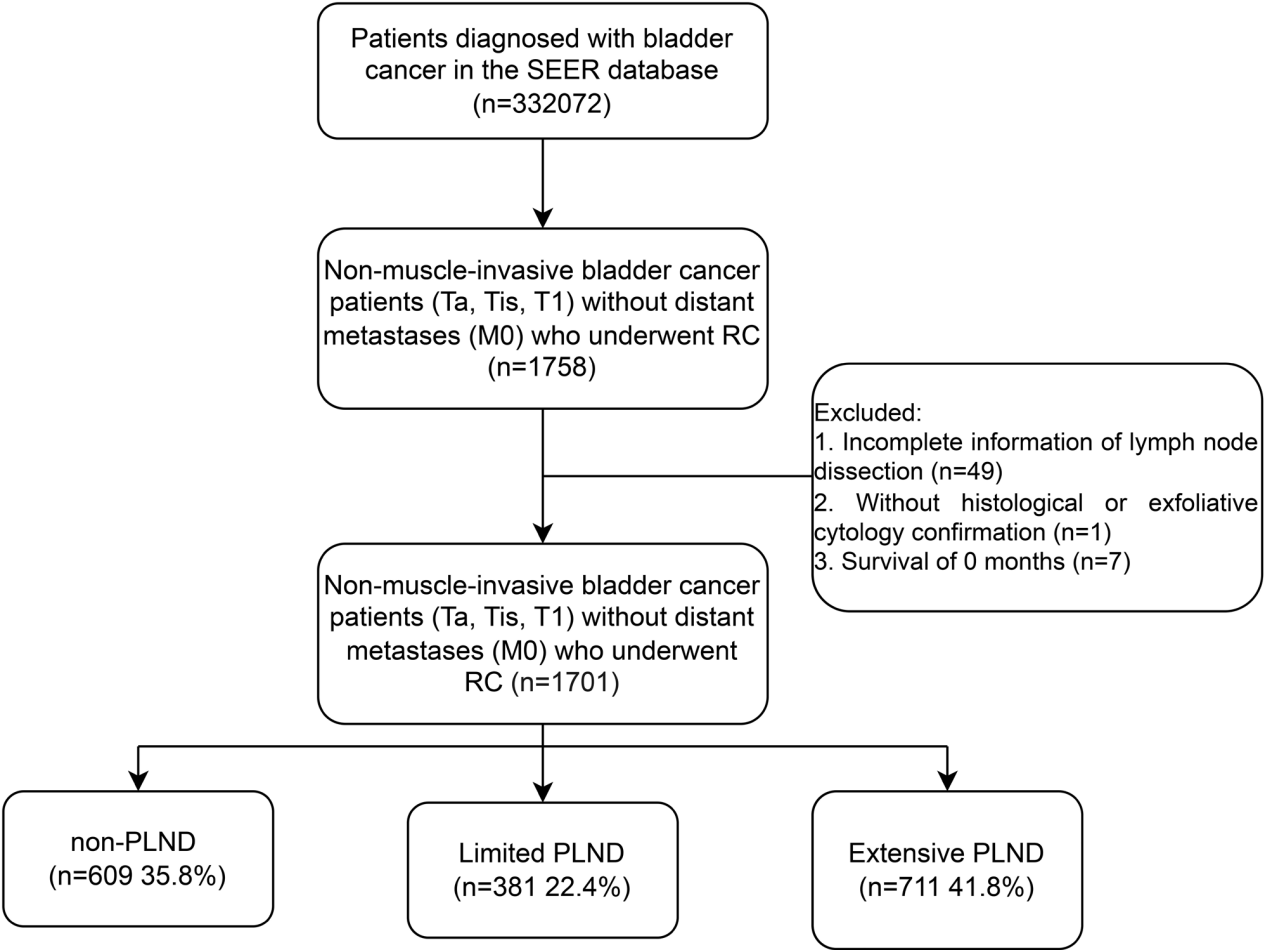
CONSORT diagram.

The National Cancer Institute's Surveillance, Epidemiology, and End Results (SEER) database, which gathers and publishes survival results for approximately 28% of the American population, provided us with data on patients who were diagnosed with NMIBC and underwent RC with or without PLND from 2004 to 2015. Our study's inclusion criteria were as follows: (1) patients with the bladder as their primary location of disease (coded as C670–679) were staged as NMIBC (Ta, Tis, T1) without distant metastases (M0); (2) patients underwent RC (coded as 50, 60–64, 80) with or without PLND. Patients with urothelial carcinoma (coded as 8050, 8120, 8130) or non-urothelial carcinoma were both included in our study. Patients without complete information on PLND (the scope of local lymph node surgery and the number of lymph nodes [LNs] examined) or microscopic confirmation (positive histology or exfoliative cytology) were excluded from our study, as were patients with a survival of 0 months. The screening process is outlined in Figure 1.

### Covariates

Demographic covariates included the patient's age (<65, ≥65 years), gender (male, female), race (white, others/unknown), and year of diagnosis (2004–2015). Tumor-related factors consisted of T (Ta, Tis, T1) and N stage (N0, N+) based on the 6th American Joint Committee on Cancer, histological type (urothelial carcinoma, non-urothelial carcinoma), and tumor grade (low grade, G1-G2; high grade, G3-G4; unknown). Patients with known tumor size were divided into two groups (<3 cm, ≥3 cm) according to EAU guidelines (3). Implementation (yes, no) and the scope of PLND were treatment-related factors. As with a previous study, we appraised the implementation of PLND based on the combination of records of the scope of local lymph node surgery and the number of LNs examined in the SEER database; only where there was an objective of PLND and the number of LNs examined was greater than 0 did we regard PLND to be conducted (7). Based on the number of LNs examined, we further divided patients who underwent PLND into the limited (1–9 LNs examined) and extensive (≥10 LNs examined) PLND groups, with a value of 10 as the cutoff (7).

### Statistical analysis

To compare patient characteristics between the PLND and non-PLND groups, the chi-square test for categorical variables and the Mann–Whitney U test for patient numerical age and the number of LNs examined were utilized. The Kaplan–Meier method was used to estimate overall survival (OS), with the log-rank test to assess the difference between the PLND and non-PLND groups. Subgroup analysis was then carried out using stratification based on predetermined demographic and oncological parameters. Next, we examined the effect of the scope of PLND by dividing patients into the non, limited, and extensive PLND groups based on the number of LNs examined (non, 0; limited, 1–9; extensive, ≥10) and plotting survival curves for overall comparison with the log-rank test. Pairwise comparisons were also performed, with the Bonferroni method adjusting the significance level. Finally, we applied Cox regression model to identify independent predictors. To identify potential predictors as much as possible, variables with *p* < 0.15 in univariate analysis were included in the multivariate analysis. All tests were two-sided, and *p* < 0.05 was considered statistically significant except in the univariate analysis of the Cox proportional hazards model and pairwise comparisons.

## Results

### Patient characteristics

From the SEER database, we enrolled 1,701 patients who satisfied the study's inclusion criteria from 2004 to 2015. A total of 1,092 patients (64.2%) received any level of PLND, of which 711 patients (65.1%) were considered to have undergone extensive PLND. The proportion of patients with any degree of PLND decreased from 60.9% in 2004 to 50% in 2015, while the proportion of extended PLND increased from 30.1% in 2004 to 36.6% in 2015. The median age was 68 (interquartile range [IQR], 60–74) years for all included patients, with 70 (IQR, 61–76) years for non-PLND patients and 67 (60–74) years for PLND patients. The majority of patients were ≥65 years (61.5%), male (89.7%), white (89.1%), and presented with stage T1 (78.8%), stage N0 (97.2%), urothelial carcinoma (94.3%), and high-grade disease (72.8%). Patients with tumor size <3 cm, ≥3 cm, and unknown tumor size accounted for 18.7%, 30.7% and 50.6%, respectively. The median number of LNs was 6 (IQR, 0–18) for all included patients, with 14 (IQR, 7–24) for the PLND and 0 (IQR, 0–0) for the non-PLND groups. The median number of LNs examined was 8 (IQR, 0–20) in T1 patients, 0 (IQR, 0–11) in Ta, and 0 (IQR, 0–14) in Tis patients. Table 1 shows the characteristics of all included patients.

**Table 1:** Demographic and oncologic characteristics of the patients.

Variables	Total (*n* = 1701)	Non-PLND (*n* = 609)	PLND (*n* = 1092)	*p*
Age Median, (25th–75th percentile)	68 (60,74)	70 (61,76)	67 (60,74)	<0.001
Age, *n* (%)				0.008
<65 years	655 (38.5)	209 (34.3)	446 (40.8)	
≥65 years	1,046 (61.5)	400 (65.7)	646 (59.2)	
Sex, *n* (%)				<0.001
Male	1,525 (89.7)	526 (86.4)	999 (91.5)	
Female	176 (10.3)	83 (13.6)	93 (8.5)	
Race, *n* (%)				0.599
White	1,516 (89.1)	546 (89.7)	970 (88.8)	
Others/unknown	185 (10.9)	63 (10.3)	122 (11.2)	
T stage, *n* (%)				<0.001
Ta	236 (13.9)	127 (20.9)	109 (10.0)	
Tis	124 (7.3)	62 (10.2)	62 (5.7)	
T1	1,341 (78.8)	420 (68.9)	921 (84.3)	
N stage, *n* (%)				<0.001
N0	1,654 (97.2)	606 (99.5)	1,048 (96.0)	
N+	47 (2.8)	3 (0.5)	44 (4.0)	
Histology, *n* (%)				0.953
Urothelial carcinoma	1,604 (94.3)	574 (94.3)	1,030 (94.3)	
Non-urothelial carcinoma	97 (5.7)	35 (5.7)	62 (5.7)	
Grade, *n* (%)				<0.001
Low (G1-G2)	233 (13.7)	124 (20.4)	109 (10.0)	
High (G3-G4)	1,239 (72.8)	374 (61.4)	865 (79.2)	
Unknown	229 (13.5)	111 (18.2)	118 (10.8)	
Tumor size, *n* (%)				<0.001
<3 cm	318 (18.7)	93 (15.3)	225 (20.6)	
≥3 cm	522 (30.7)	147 (24.1)	375 (34.3)	
Unknown	861 (50.6)	369 (60.6)	492 (45.1)	
Year of diagnosis, *n* (%)				0.646
2004–2009	993 (58.4)	360 (59.1)	633 (58.0)	
2010–2015	708 (41.6)	249 (40.9)	459 (42.0)	
Number of LNs examined				
Median, (25th–75th percentile)	6 (0,18)	0 (0,0)	14 (7,24)	<0.001

PLND, Pelvic lymph node dissection.

### Overall survival

**Figure 2:**
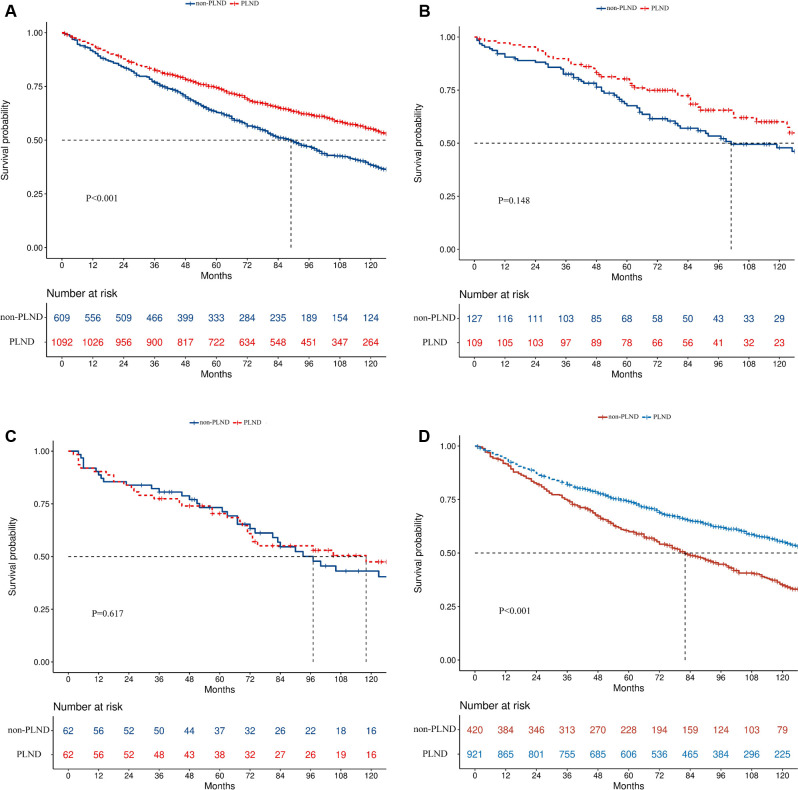
Kaplan–Meier survival curves in the complete cohort (**A**), Ta (**B**), Tis (**C**), and T1 (**D**) disease cohorts.

The median follow-up time for all included patients was 77 months (IQR, 42–116 months), with 84 months for PLND (IQR, 47–108 months) and 67 months for non-PLND patients (IQR, 37–108 months). Patients who received PLND during RC had better overall survival (OS) than those who did not (median survival time, 139 vs. 89 months, *p* < 0.001, Figure 2A).

**Figure 3:**
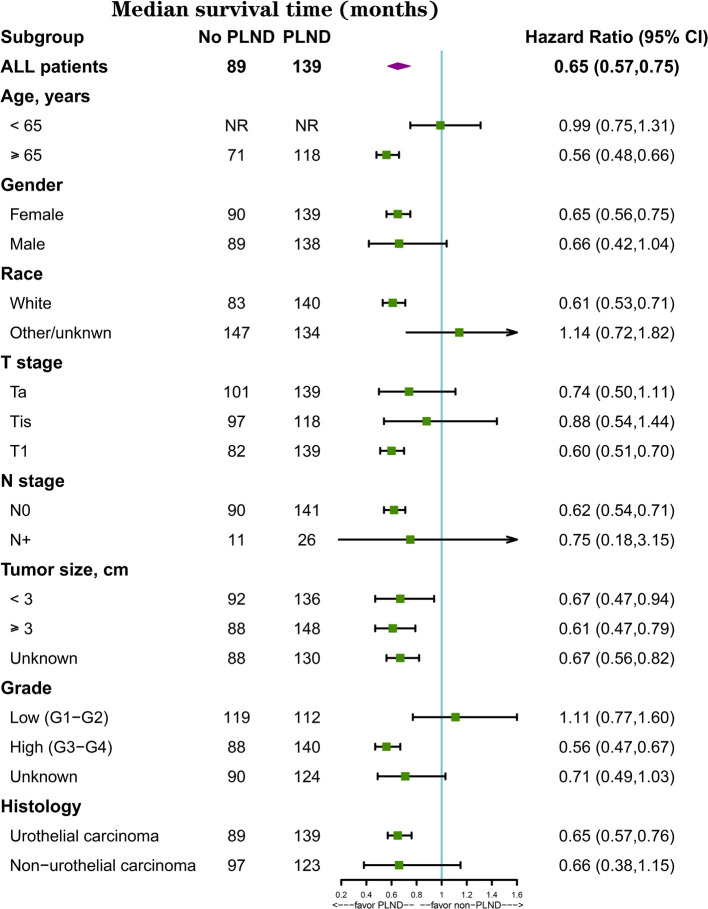
Effect of PLND in all prespecified subgroups.

In the subgroup analysis, PLND led to better OS in T1 (median survival time, 139 vs. 82 months, *p* < 0.001, Figure 2D) but not in Ta (median survival time: 139 vs. 101 months, *p* = 0.148, Figure 2B) or Tis patients (median survival time: 118 vs. 97 months, *p* = 0.617, Figure 2C). PLND also led to better OS in N0 (median survival time, 141 vs. 90 months, *p* < 0.001) but not in N+ patients (median survival time: 26 vs. 11 months, *p* = 0.698). When patients were divided according to the size of the tumor, better OS was found in both patients with tumor < 3 cm (median survival time: 136 vs. 92 months, *p* = 0.022) and ≥3 cm (median survival time: 148 vs. 88 months, *p* < 0.001). Significant OS improvement was also discovered in patients with high-grade disease (median survival time, 140 vs. 88 months, *p* < 0.001) but not in patients with low-grade disease (median survival time, 112 vs. 119 months, *p* = 0.577). In the urothelial carcinoma group (median survival time, 139 vs. 89 months, *p* < 0.001) but not in the non-urothelial carcinoma group (median survival time, 123 vs. 97 months, *p* = 0.139), the effect of PLND was significant. The forest plot depicts all the results of survival analyses for predetermined subgroups (Figure 3).

**Figure 4:**
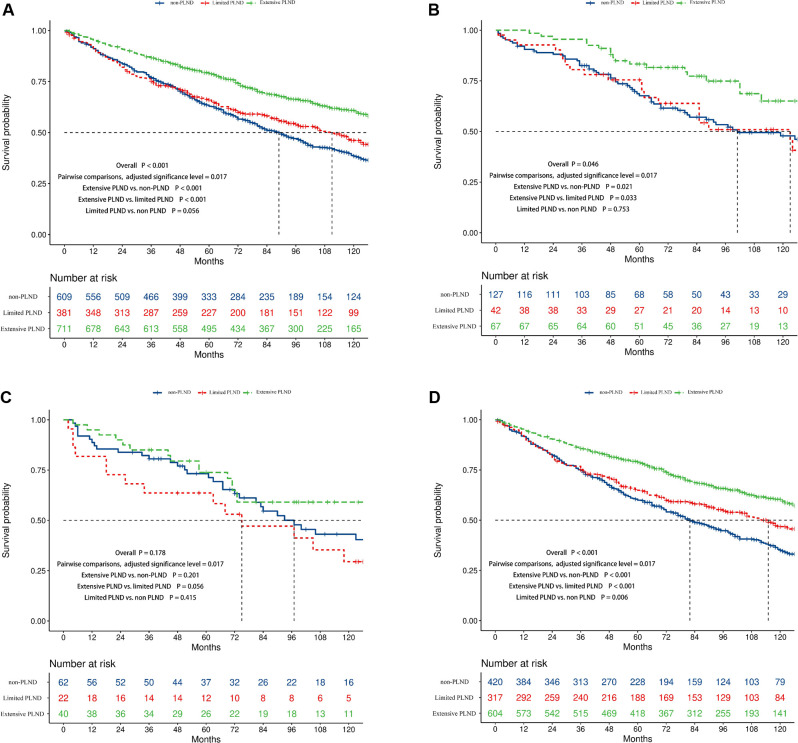
Kaplan–Meier survival curves showing the survival difference between the non, limited, and extensive PLND patients in the complete cohort (**A**), Ta (**B**), Tis (**C**), and T1 (**D**) disease cohorts.

When patients were separated into extensive, limited, and non-PLND groups based on the number of LNs examined, the survival difference was significant in all included (median survival time, 151 vs. 111 vs. 89 months, *p* < 0.001, Figure 4A), Ta (median survival time, not reached vs.123 vs. 101 months, *p* = 0.046, Figure 4B), and T1 patients (median survival time, 150 vs. 115 vs. 82 months, *p* < 0.001, Figure 4D) but not in Tis patients (median survival time, 158 vs.75 vs. 97 months, *p* = 0.178, Figure 4C). The adjusted significance level was 0.017 in pairwise comparisons. Compared to limited and non-PLND, extensive PLND resulted in better OS in T1 (Extensive PLND vs. non-PLND, *p* < 0.001, Extensive PLND vs. limited PLND, *p* < 0.001, Figure 4D) but not in Ta (Extensive PLND vs. non-PLND, *p* = 0.021, Extensive PLND vs. limited PLND, *p* = 0.033, Figure 4B) or Tis patients (Extensive PLND vs. non-PLND, *p* = 0.201, Extensive PLND vs. limited PLND, *p* = 0.056, Figure 4C). Compared with non-PLND, limited LND achieved a better OS in T1 (*p* = 0.006, Figure 4D) but not in Ta (*p* = 0.753, Figure 4B) or Tis patients (*p* = 0.415, Figure 4C).

The results of univariate and multivariate Cox regression analyses are shown in Table 2. Age, T stage, N stage, histology, and PLND were then included in the multivariate Cox regression analysis after univariate Cox regression analysis (all *p* < 0.15). In the multivariable Cox analysis, PLND (non-PLND as a reference, limited PLND, HR = 0.78, 95% confidence interval [CI] 0.65–0.93, P= 0.006; extensive PLND, HR = 0.53, 95% CI, 0.45–0.62, *p* < 0.001) was identified as an independent protective factor for survival. Age ≥65 years (<65 years as a reference, HR = 1.95, 95% CI, 1.67–2.28, *p* < 0.001) and N+ (N0 as a reference, HR = 3.59, 95% CI, 2.55–5.04, *p* < 0.001) were independent risk factors for survival.

**Table 2:** Univariate and multivariate regression analyses for overall survival in patients diagnosed with NMIBC who underwent RC.

Variables	Univariate		Multivariate	
	HR (95% CI)	*p*	HR (95% CI)	*p*
Age, years				
<65	reference		reference	
≥65	2.00 (1.71,2.33)	<0.001	1.95 (1.67,2.28)	<0.001
Sex				
Male	reference			
Female	1.07 (0.84,1.36)	0.579		
Race				
White	reference			
Others/unknown	0.92 (0.73,1.17)	0.505		
T stage				
Ta	reference		reference	
Tis	1.309 (0.96,1.79)	0.093	1.174 (0.86,1.61)	0.317
T1	1.165 (0.94,1.44)	0.16	1.233 (0.99,1.53)	0.058
N stage				
N0	reference		reference	
N+	3.07 (2.19,4.29)	<0.001	3.59 (2.55,5.04)	<0.001
Histology				
Urothelial carcinoma	reference		reference	
Nonurothelial carcinoma	1.26 (0.95,1.68)	0.111	1.163 (0.87,1.55)	0.304
Tumor size				
<3 cm	reference			
≥3 cm	1.05 (0.85,1.29)	0.659		
Unknown	1.11 (0.92,1.35)	0.273		
Grade				
Low (G1-G2)	reference			
High (G3-G4)	0.97 (0.80,1.19)	0.786		
Unknown	1.08 (0.84,1.40)	0.548		
PLND				
Non-PLND	reference		reference	
Limited PLND	0.84 (0.71,1.00)	0.051	0.78 (0.65,0.93)	0.006
Extensive PLND	0.56 (0.47,0.65)	<0.001	0.53 (0.45,0.62)	<0.001

HR, hazard ratio; CI, confidence interval; PLND, pelvic lymph node dissection.

## Discussion

Lymph node dissection has been shown to play a role in a variety of primary tumors, including prostate (17), testicular (18), penile (19), breast (20), colon cancer (21), etc. However, the effect and the optimal scope of PLND for NMIBC patients remain controversial. In a previous SEER database-based study, it was reported that PLND resulted in better survival in both Ta-Tis and T1 patients (10). In a study that included 114 patients, the survival benefit of PLND was stage specific and limited to Tis and T1 patients (9). In our study, the OS benefit of any degree of PLND was only observed in T1 patients. This may be due to the stage-specific lymph node metastasis rate, with a decreased rate of lymph node metastases in Ta-Tis and as high as 40% rate of lymph node metastases in T1 patients; thus, T1 patients were more likely to benefit from PLND. Regarding the impact of the extent of PLND on survival, we found that compared to non and limited PLND, extensive PLND resulted in better OS in T1 patients but not in Ta and Tis patients. Similar to our study, it was found that extensive PLND (LNs examined ≥10) was associated with better OS and the effect was limited to T1 patients in a study based on the national cancer database (7). It was also found that lymph node yield (LNY) >10 resulted in better local pelvic recurrence-free survival compared to LNY ≤10, and LNY >20 improved cancer-specific survival (CSS) and OS compared to LNY ≤20 in Tis and T1 patients in a multi-institutional retrospective study that included 1,647 patients (8). In contrast, James et al. showed no impact of PLND extent (≥10 nodes removed) on recurrence-free survival (RFS) in patients diagnosed with NMIBC (6). Similarly, in a prospective randomized trial including 401 patients diagnosed with locally resectable T1G3 or MIBC, extensive PLND (based on the anatomy) was unable to achieve significant improvements in RFS, CSS, and OS relative to limited PLND (11). Unexpectedly, PLND did not improve OS in patients with lymph node metastasis in our study. However, this may be affected by the limited sample amount. Overall, there were only 47 (2.8%) patients with lymph node metastasis in the included population. Taken together, these conflicting results suggest that large, prospective studies are needed to elucidate the impact of PLND during RC on survival for patients diagnosed with NMIBC.

Shariat et al. concluded that at least 6 LNs for Ta-Tis patients and 9 for T1 patients should be eliminated after reviewing over 4,000 patients who underwent RC (22). In this study, 1,092 out of 1,701 patients (64.2%) received any level of PLND, of which only 711 patients (65.1%) were considered extensive in our study. The median number of LNs examined was 8 (IQR 0–20) for T1 patients, 0 (IQR 0–11) for Ta and 0 (IQR 0–14) for Tis patients. Both the proportion and scope of PLND seemed to be insufficient. The limited implementation of PLND may be due to surgeon uncertainty about the effect of PLND and the greater surgical difficulty of PLND during RC.

Our research has a few drawbacks. First, this is a retrospective study based on the SEER database, which has its own set of limitations. Second, more LNs removed indicated more thorough resection, which is more common among more experienced and better-trained physicians and may result in better survival outcomes, which may lead to bias. Additionally, we were unable to conduct subgroup analyses based on patient performance status and comorbidities, which are vital to patient survival, because detailed information is not available in the SEER database. Finally, we assessed the scope of LND based on the number of lymph nodes examined, which may vary depending on a variety of parameters, including interindividual variability and pathologic thoroughness. As a result, if dissection is careful, the LN count is less relevant than the anatomical LND template, which is not available.

## Conclusion

Based on the SEER database, we found that PLND during RC led to better OS and extensive PLND was associated with better OS in T1 but not in Ta or Tis patients. The implementation of PLND was insufficient both in population proportions and scope.

## Data Availability

The original contributions presented in the study are included in the article/Supplementary Material, further inquiries can be directed to the corresponding author/s.
